# Investigation on the Interactions between Self-Assembled β-Sheet Peptide Nanofibers and Model Cell Membranes

**DOI:** 10.3390/ijms21249518

**Published:** 2020-12-14

**Authors:** Tomonori Waku, Ayane Kasai, Akio Kobori, Naoki Tanaka

**Affiliations:** Faculty of Molecular Chemistry and Engineering, Kyoto Institute of Technology, Gosyokaido-cho, Matsugasaki, Sakyo-ku, Kyoto 606-8585, Japan; ayn.chacha@hotmail.co.jp (A.K.); akobori@kit.ac.jp (A.K.); tanaka@kit.ac.jp (N.T.)

**Keywords:** nanofiber, DPPC Langmuir membrane, π–*A* isotherm, drug delivery system

## Abstract

Self-assembled peptide nanofibers (NFs) obtained from β-sheet peptides conjugated with drugs, including antigenic peptides, have recently attracted significant attention. However, extensive studies on the interactions of β-sheet peptide NFs with model cell membranes have not been reported. In this study, we investigated the interactions between three types of NFs, composed of PEG-peptide conjugates with different ethylene glycol (EG) lengths (6-, 12- and 24-mer), and dipalmitoylphosphatidylcholine (DPPC) Langmuir membranes. When increasing the EG chain length, those interactions significantly decreased considering measurements in the presence of the NFs of: (i) changes in surface pressure of the DPPC Langmuir monolayers and (ii) surface pressure–area (π–*A*) compression isotherms of DPPC. Because the observed trend was similar to the EG length dependency with regard to cellular association and cytotoxicity of the NFs that was reported previously, the interaction of NFs with phospholipid membranes represented a crucial factor to determine the cellular association and toxicity of the NFs. In contrast to NFs, no changes were observed with varying EG chain length on the interaction of the building block peptide with the DPPC membrane. The results obtained herein can provide a design guideline on the formulation of β-sheet peptide NFs, which may broaden its potential.

## 1. Introduction

Nano-sized materials have been widely used for biomedical applications, such as drug delivery systems [1]. The intrinsic surface properties of these materials, such as charge and hydrophobicity, affect their interaction with cellular membranes [2]. The interaction of nanomaterials with the cellular membrane is one of the most important factors that determine their cellular uptake, cytotoxicity, and cellular response [3]. Thus, in nanomaterials’ design with specific properties according to their applications, the understanding of the interactions between nanomaterials and cell membranes is important [4,5]. However, it is difficult to study these interactions, owing to the complexity of the cellular membrane. Using a simple membrane model, such as supported lipid bilayers [6,7], liposome membranes [8], and Langmuir lipid monolayers [9,10,11,12,13,14,15,16,17,18,19,20,21,22,23,24,25,26,27,28], is an effective approach. Among these, Langmuir monolayers containing lipids is one of the commonly used systems to study the interactions between nanomaterials and model cell membranes [17,18,19,20,21,22,23,24,25,26,27,28]. The surface pressure of the Langmuir monolayer is related to the molecular lateral packing of lipids in the monolayer [29], and it is thus influenced by interactions between the nanomaterials and lipids. Therefore, surface pressure measurements in the lipid monolayer under the presence of a nanomaterial can provide information on the interactions between them.

Considerable effort has been made to develop nanoparticles-based delivery systems for drugs (including peptides, proteins, and nucleic acids) through various types of nanoparticles, such as liposomes [30], polymeric nanoparticles [31], and polymeric micelles [32]. Although these systems are useful, most of them need multi-step complicated procedures with respect to synthesis of the constituent molecules, preparation of the nanoparticles, and drug loading. Moreover, their drug-loading efficiency and the types of drugs that can be used are limited. Recently, self-assembly approach using β-sheet forming peptides conjugated with drugs (e.g., anti-cancer drugs, peptide drugs, and antigenic peptides) has attracted attention as a simpler method to prepare nanoformulations [33,34,35,36,37]. The self-assembling method is advantageous because (i) a β-sheet-based fibrous assembly can provide highly efficient drug loading without laborious procedures; (ii) the resulting nanofibers (NFs) are composed of a single molecule without molecular weight distribution unlike polymer molecules, and (iii) the building block peptides can be synthesized relatively easily and have high biocompatibility. However, fundamental studies on the interactions of self-assembled β-sheet peptide NFs with phospholipid membranes have not been reported. Typically, β-sheet peptides assemble into NFs forming a cross-β sheet structure [38]. Because such molecular assembling is not as simple as the self-assembling of amphiphilic molecules to micelles (which possess a hydrophobic core uniformly covered with hydrophilic segments), it is difficult to predict the interactions of NFs with the cell membrane only from the molecular structure of the building block. Therefore, a detailed investigation on how the molecular structures (e.g., hydrophilic–hydrophobic balance) of the NFs affect their interactions with the cell membrane is important to broaden potential applications of the β-sheet peptide NFs.

**Figure 1:**
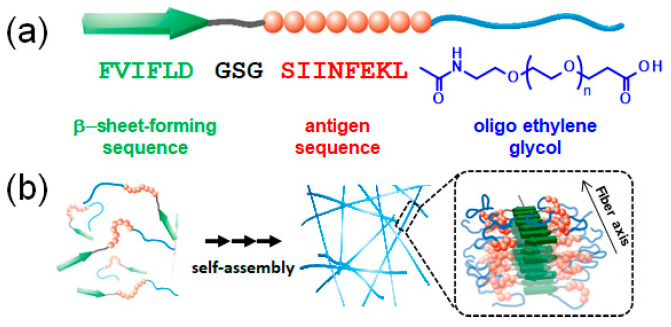
(**a**) Design of the building block peptides (EG_n_) composed of a β-sheet-forming sequence (FVIFLD), a flexible-linker block (GSG), a model antigen sequence (SIINFEKL from Ovalbumin), and oligo(ethylene glycol); (**b**) schematic of the self-assembling process for nanofiber formation and the proposed model of highly antigen-loaded nanofibers based on a study previously reported [39].

In a previous study, we have reported the preparation of antigenic peptide-loaded NFs by using self-assembled β-sheet peptides conjugated with model antigen peptides from ovalbumin and oligo(ethylene glycol) (EG) for antigenic peptide delivery (Figure 1) [36,37,39,40]. Therein, the effect of EG length on the cellular association and toxicity of the NFs was investigated, and we had observed that those with shorter EG length exhibited both higher cellular association and toxicity [40]. However, the EG length-dependency on their cellular association and cytotoxicity are well not understood in terms of the interactions between NFs and cell membranes. If the relationship between the cellular system and model membrane system would be elucidated by fundamental investigation using Langmuir model membrane, that would provide one design guideline of functional drug-conjugated peptide nanofibers for intracellular delivery.

In this study, we investigated the interactions between NFs (three types with different EG lengths) and dipalmitoylphosphatidylcholine (DPPC) Langmuir monolayers, which are frequently used on determining the interactions of nanomaterials and model biomembranes [9,20,21,22,23,26,27,28]. The timely changes in the surface pressure of the pre-prepared DPPC-Langmuir monolayers due to the interaction with the NFs, and surface pressure–Area (π–*A*) isotherms of DPPC in the presence of NFs were measured. From these results, it was derived that increasing the EG chain length significantly decreased the interactions with the DPPC membrane, which may be correlated with their cellular association and toxicity.

## 2. Results

### 2.1. Interactions of NFs with the DPPC Membrane

**Figure 2:**
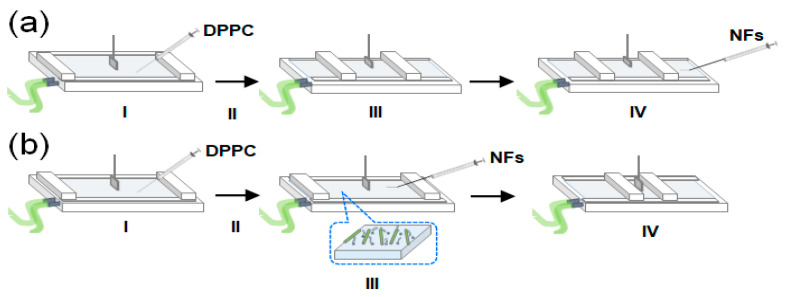
Schematic of the experimental procedure: (**a**) change in surface pressure after the injection of EG_n_ NFs into the subphase; (I) spread of DPPC in chloroform on phosphate buffered saline (PBS) subphase using a micro-syringe, (II) chloroform evaporation, (III) prefabrication of DPPC membrane with a surface pressure of ~30 mN/m, and (IV) injection of NFs into the subphase using a micro-syringe; (**b**) π–*A* compression isotherm of DPPC in the presence of NFs; (I) spread of DPPC in chloroform on the PBS subphase using a micro-syringe, (II) chloroform evaporation, (III) injection of NFs into the subphase using a micro-syringe, and (IV) compression of the DPPC membrane at a speed of 2 mm/min.

**Figure 3:**
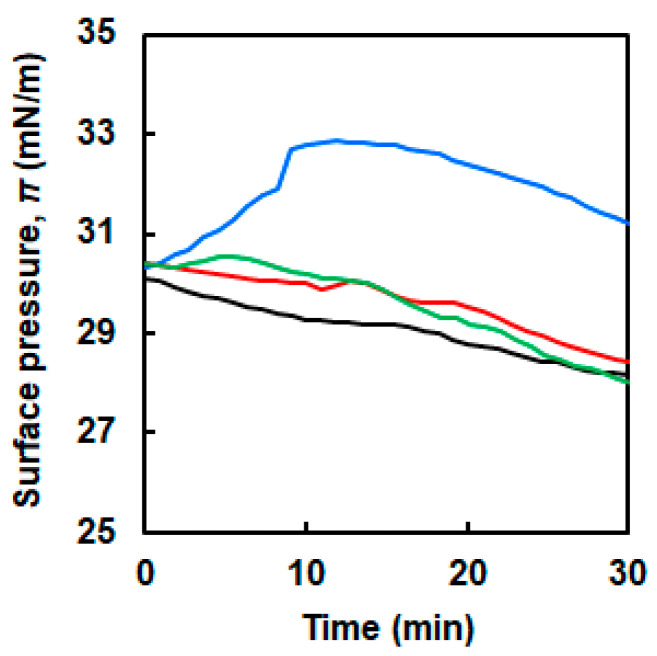
Changes in surface pressure of the DPPC membrane as a function of time in the absence of (black line) and presence of the EG_n_ NFs; *n* = 6 (blue line), 12 (red line), 24 (green line).

Changes in surface pressure with time under a constant trough area were recorded to investigate the interaction between NFs and the DPPC membrane (Figure 2a). A DPPC membrane with a surface pressure of ~30 mN/m was prefabricated. The three types of NFs with different EG lengths (6-, 12- and 24-mer; denoted as EG_6_, EG_12_, and EG_24_, respectively) were slowly injected into the subphase of the DPPC membrane. Then, changes in surface pressure for 30 min were measured. Because the lateral pressure of the human cell membranes is reported to be ~30 mN/m, this value was set as the initial surface pressure [41]. In the absence of NFs, the surface pressure of the DPPC membrane slightly decreased (Figure 3, Figure S1). When EG_12_ NFs and EG_24_ NFs were added, the change in surface pressure was similar to that of the DPPC membrane. On the other hand, when EG_6_ NFs were added, the surface pressure significantly increased compared to the initial value.

### 2.2. Effect of NFs on the π–A Isotherm of the DPPC Membrane

**Figure 4:**
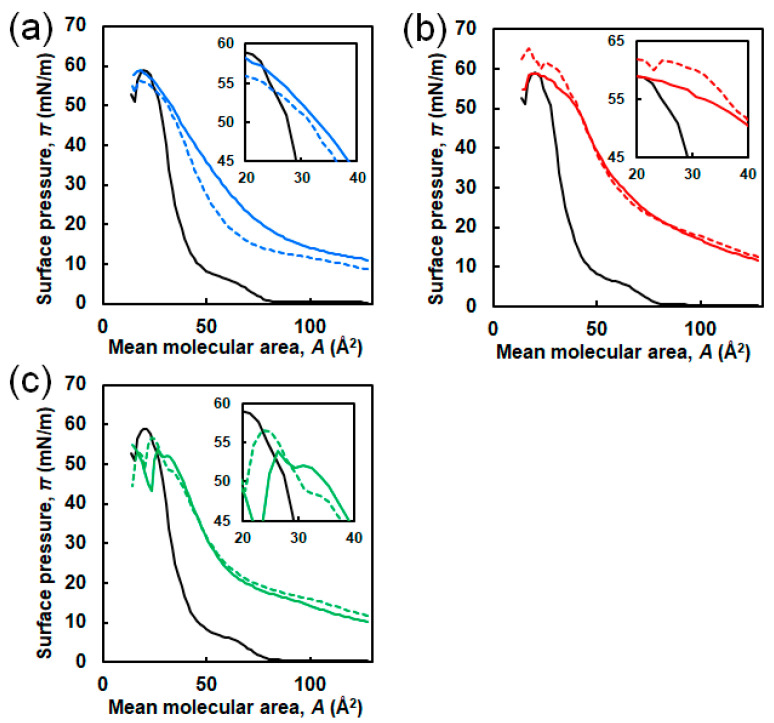
π–*A* isotherms of DPPC in the presence of (**a**) EG_6_ NFs, (**b**) EG_12_ NFs, and (**c**) EG_24_ NF. The concentrations of NFs were 1.0 μM (colored dashed line) and 2.5 μM (colored solid line). Black lines represent π–*A* isotherms of sole DPPC. The inset is an enlarged view in the range of 20–40 Å^2^/molecule.

To evaluate the interaction of EG_n_ NFs with Langmuir DPPC membranes, π–*A* compression isotherms of DPPC were obtained in the presence of EG_n_ NFs, previously injected into the subphase (Figure 2b). The compression isotherm of sole DPPC obtained upon continuous unilateral compression at a speed of 2 mm/min and *T* = 22 °C is shown in Figure 4 (black solid line). The shape of the curve was approximately consistent with previously reported data [42,43,44]. Four distinctive curve regions were observed. Analyzing the isotherms from right to left, the value of surface pressure was zero in the region from *A* ≈ 128 Å^2^/molecule to A ≈ 85 Å^2^/molecule (first plateau in the *x*-axis), and then increased linearly. Then, followed by near-horizontal region, the steep increase in π were observed. Based on data from the literature, the four regions can be attributed to the G (gas)-LE (liquid expanded), the LE, the LE-LC (liquid condensed), and the LC phases, respectively (*x*-values from right to left) [43]. In addition, a kink at *A* ≈ 29 Å^2^/molecule corresponding to π ≈ 52 mN/m was observed. This point was attributed to DPPC-monolayer collapse caused by failure to withstand further compression.

The compression π–*A* isotherms of DPPC in the presence of EG_n_ NFs are shown in Figure 4 (colored line). Collapse surface pressures and limiting molecular areas are summarized in Table S1. The concentrations of NFs were 1.0 μM (dashed line) and 2.5 μM (solid line). It should be noticed that the mean molecular area (*A*) was calculated dividing the trough area by the number of DPPC molecules without considering peptide molecules. This value represented the apparent molecular area of the DPPC molecule at the air–water interface. In the presence of NFs, π values at initial compression (i.e., when *A* ≈ 128 Å^2^/molecule) were positive irrespective of the EG length. Moreover, the isotherm curves of DPPC in the presence of NFs shifted toward higher *A* compared to sole DPPC. The calculated Δ*A*, which was defined as the difference between the *A* value of the sole DPPC monolayer and the *A* value of the DPPC/NFs membrane, is suggestive about the interaction between NFs and DPPC. In the case of EG_24_ NFs, Δ*A* decreased with increasing π and was nearly zero (≤ 2 Å^2^/molecule) for *A* values in the range of 25–31 Å^2^/molecule, i.e., the isotherm curves of the binary system almost overlapped with those of sole DPPC (Figure 4c inset). On the other hand, in the case of EG_6_ NFs and EG_12_ NFs, Δ*A* was positive until the intersection of the curve of sole DPPC and that of binary system.

To understand the effect of NFs on the DPPC monolayer, the elastic moduli (*ε*_0_) were calculated from the π–*A* isotherm curves based on the following equation.

(1) $$\varepsilon_{0}=-A\left(\frac{\delta \pi }{\delta A}\right)$$

**Figure 5:**
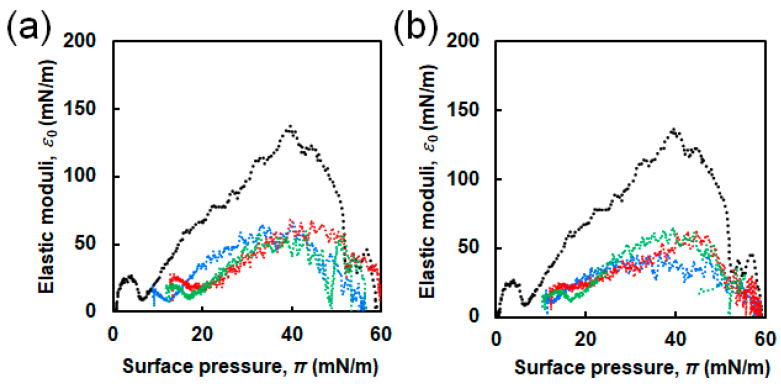
Elastic modulus (*ε*_0_) as function of surface pressure (π) for DPPC in the presence of EG_n_ NFs at different concentrations: (**a**) 1.0 µM and (**b**) 2.5 µM; *n* = 6 (blue line), 12 (red line), 24 (green line). Black lines represent *ε*_0_–π plots for sole DPPC.

*ε*_0_ values are important because they are related to the rigidity and elastic properties of the monolayer [42]. The obtained *ε*_0_ values were plotted as a function of π (Figure 5). For sole DPPC, the two maximal points were observed at π of approximately 4 and 40 mN/m, corresponding to *ε*_0_ of ≈26 and 137 mN/m, which were attributed to the LE and LC phases, respectively [27]. The convex curves for the binary systems of DPPC and EG_n_ NFs were less sharp than that of sole DPPC. The estimated maximum *ε*_0_ values are summarized in the bottom row of Table S1. The presence of NFs caused a significant decrease in the maximum *ε*_0_ values irrespective of the EG length.

### 2.3. Interaction of EG_n_ Peptides with the DPPC Membrane

**Figure 6:**
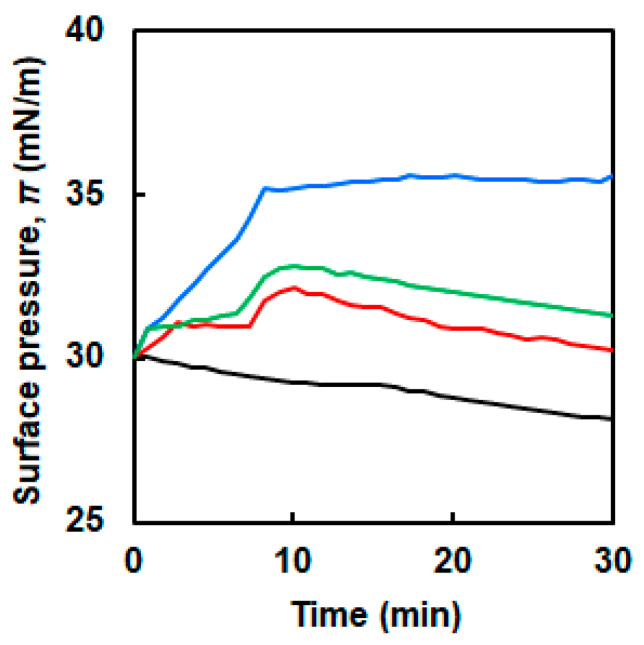
Changes in surface pressure of the DPPC membrane as a function of time in the absence of (black line) and presence of the EG_n_ peptides; *n* = 6 (blue line), 12 (red line), 24 (green line).

To gain insight to the significance of nanofiber structures regarding their interaction with the DPPC membrane, similar measurements using non-fibrillated peptides (EG_6_ peptide, EG_12_ peptide, and EG_24_ peptide) were performed. Figure 6 shows π values in the DPPC membrane after injection of the EG_n_ peptides. For the three EG_n_ peptide samples, π gradually increased over time. In particular, the addition of the EG_6_ peptide caused a more significant increase in π compared to the other peptides.

### 2.4. Effect of EG_n_ Peptides on the π–A Isotherm of the DPPC Membrane

**Figure 7:**
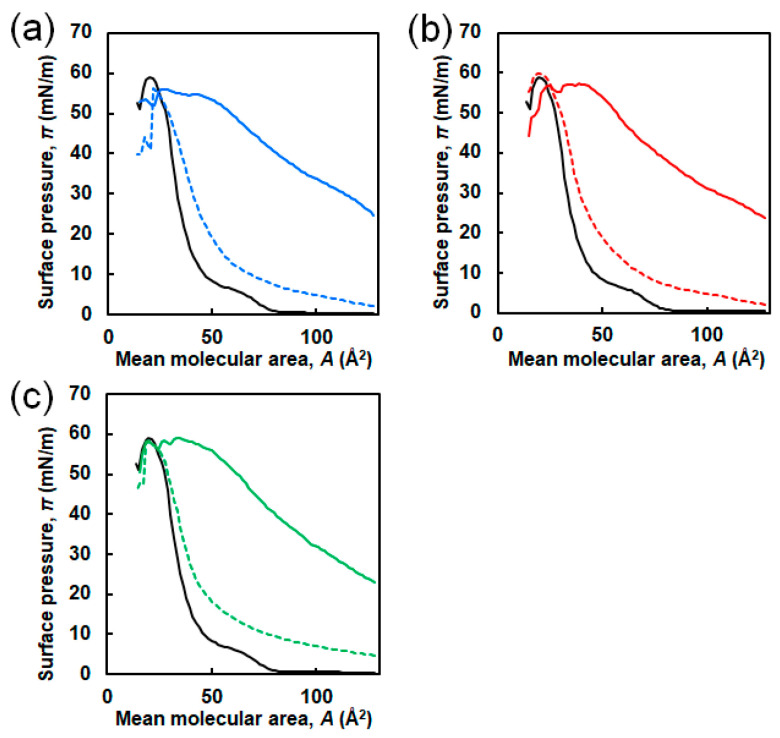
π–*A* isotherms of DPPC in the presence of (**a**) EG_6_ peptides, (**b**) EG_12_ peptides, and (**c**) EG_24_ peptides. The concentrations of peptides were 0.1 μM (colored dashed line) and 1.0 μM (colored solid line). Black lines represent π–*A* isotherms of sole DPPC.

**Figure 8:**
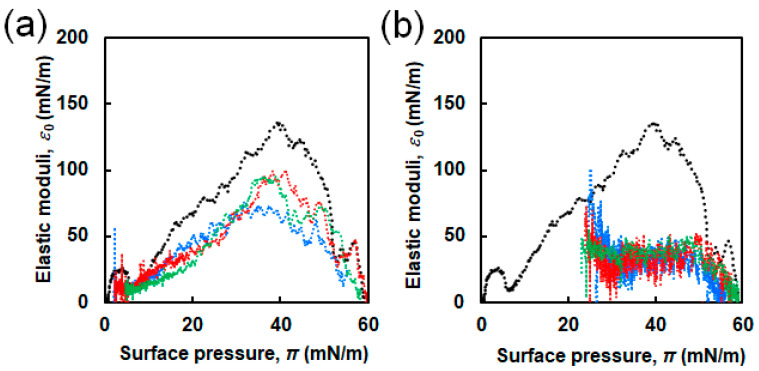
Elastic modulus (*ε*_0_) as function of surface pressure (π) for DPPC in the presence of EG_n_ peptides at different concentrations: (**a**) 0.1 µM and (**b**) 1.0 µM; *n* = 6 (blue line), 12 (red line), 24 (green line). Black lines represent *ε*_0_–π plots for sole DPPC.

Figure 7 shows the π–*A* isotherm curves of DPPC in the absence and presence of three types of EG_n_ peptides at different concentrations (0.1 and 1.0 μM). Although no significant difference due to the EG length was observed in the π–*A* isotherms (unlike the EG_n_ NFs/DPPC systems), highly different curves were obtained at different peptide concentrations. π values at initial compression were approximately 2–5 and 22–25 mN/m for 0.1 and 1.0 μM EG_n_ peptides, respectively. Comparing with the EG_n_ NFs systems at the same concentration (1.0 μM), the initial π value in the presence of EG_n_ peptides was much higher. The isotherms in the presence of EG_n_ peptides at a concentration of 0.1 μM shifted toward higher *A* compared to the isotherm of DPPC. However, they almost overlapped with the isotherm of DPPC in the region of higher π. On the other hand, when the peptide concentration was 1.0 μM, the isotherms of the EG_n_ peptide/DPPC system entirely differed from those of sole DPPC. Figure 8 shows the *ε*_0_-π plot from π–*A* isotherm calculations. When the peptide concentration was 0.1 μM, the *ε*_0_–π curves were similar to that of sole DPPC. The maximum *ε*_0_ values were 75, 95, and 100 mN/m for the EG_6_-peptides, EG_12_-peptides, and EG_24_-peptides samples, respectively. When the peptide concentration was 1.0 μM, the *ε*_0_-π curves exhibited clear differences from that of sole DPPC.

## 3. Discussion

Previously, we stated that the EG length of NFs significantly influences their cellular association and toxicity [40]. For further understanding on the effect, we investigated herein the interactions between the DPPC Langmuir membrane and three types of NFs with different EG lengths (6-, 12- and 24-mer).

The changes in π with time after the injection of EG_n_ NFs below the membrane differed significantly according to the EG length (Figure 3). The addition of EG_6_ NFs caused an increase in π. On the other hand, when EG_12_ NFs and EG_24_ NFs were added, π variations were similar to those for the sole DPPC membrane. Typically, an increase in π upon injection of some additives, such as polymers and nanomaterials, to the subphase can be attributed to lipid molecules condensation due to lipid–additive interactions. Two possible scenarios in which phospholipid packing can be more condensed due to the interaction with additives are described as follows: (i) the additives interact electrostatically with phospholipid molecules, leading to reduction in the repulsive forces between the lipid molecules [11,12]; (ii) the additives incorporate to the water–air interface via hydrophobic interactions with the hydrophobic tail of the phospholipids [13,25,41]. These interactions can lead to a decrease in the phospholipids’ available area and an increase in π. Moreover, when π remains constant, no interactions exist between the additives and the membrane [14,15]. Based on these findings, it can be derived that EG_6_ NFs exhibited a stronger interaction with the DPPC membrane compared to EG_12_ NFs and EG_24_ NFs. Possible interactions between the membrane and EG_6_ NFs will be further discussed below.

**Figure 9:**
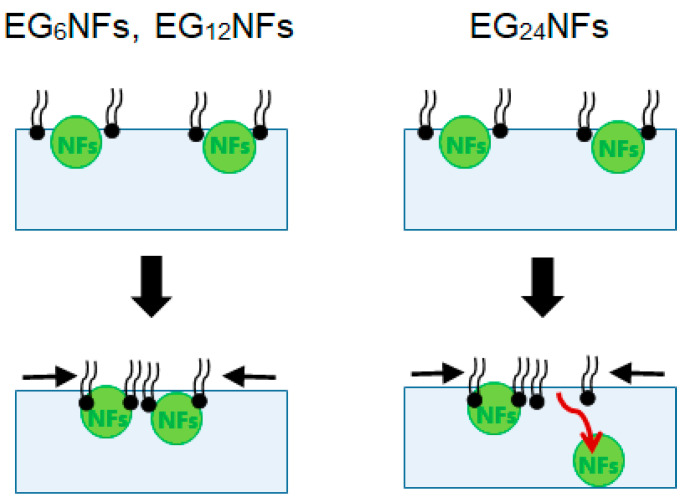
Schematic of the sequence of interactions between the DPPC monolayers and the EG_n_ NFs at the air–water interface.

In the π–*A* isotherms for the binary system and the sole DPPC membrane, three differences were observed, suggesting that the EG_n_ NFs influence on the lateral packing of DPPC monolayers and their mechanical characteristics (Figure 4). First, π values at zero compression were positive in the presence of EG_n_ NFs, irrespective of the EG length. This was related to the adsorption of NFs at the air–water interface, owing to their amphiphilic properties, which can be easily speculated from their composition. Second, the isotherms for the DPPC/EG_n_ NFs systems shifted toward higher *A* value compared to that for sole DPPC. As *A* represents the apparent mean area *per* phospholipid molecule (trough area divided by the number of DPPC molecules), tracing the π-*A* isotherm from right to left demonstrates an increase in π with compression. Thus, the observed shift toward higher *A* indicated that π for the NFs-containing system exhibited higher increases at lower degrees of compression compared to the sole DPPC membrane. The shift toward higher *A* is typically attributed to the contribution of external additives adsorbed at the air–water interface. Based on these findings, our results can be interpreted as follows. In the presence of EG_24_ NFs, the value of Δ*A* was positive in the range of low π (≤49–52 mN/m). However, in the region where π increased up to 49–52 mN/m and the membrane collapsed, Δ*A* was closed to zero (namely two isotherms were almost overlapped) (Figure 4c). EG_24_ NFs at the air–water interface at the initial point of compression would have associated with a DPPC molecule in the plane of the monolayer, at relatively low π. However, when π increased with further compression, EG_24_ NFs would squeeze out to the subphase, indicating little interaction between DPPC and EG_24_ NFs (Figure 9, right). A similar pattern for π–*A* isotherms of phospholipid membranes in the presence of different additives (including hydrophilic nanoparticles [28], polymers [15], and peptides [9]) has been reported. In contrast, when EG_6_ NFs and EG_12_ NFs were added, the value of Δ*A* was positive within almost the entire range of π (Figure 4a,b). These results indicate that EG_12_ NFs and EG_6_ NFs were retained in the plane of the monolayer without desorption, even in the range of higher π (Figure 9, left). A similar π–*A* isotherm in the presence of hydrophobic nanoparticles had been reported [21,25,26]. Third, the *ε*_0_-π plot demonstrated that NFs caused a significant decrease in the maximum *ε*_0_ value irrespective of EG length (Figure 5). Compressing the DPPC membrane in the presence of NFs eventually created an interfacial layer between NFs and DPPC, preventing packing between DPPC molecules. This might have led to a decrease in the rigidity of the membrane. Importantly, the maximum *ε*_0_ for EG_6_ NFs was the smallest among the EG_n_ NFs systems, which indicated that the disturbing effect of EG_6_ NFs were the greatest (Table S1).

The change in π due to NFs addition indicated that the interaction of EG_6_ NFs with the DPPC membrane was the strongest among the EG_n_ NFs systems (Figure 3). The π–*A* isotherms demonstrated that the EG_24_ NFs interaction with DPPC molecules was the lowest (Figure 4). In addition, the *ε*_0_–π plots indicated that EG_6_ NFs had a more significant influence on the DPPC monolayer packing (Figure 5). Based on the three types of systems, the interactions of NFs with the DPPC membrane were estimated to be higher with decreasing EG length. One of the possible interactions of NFs with DPPC was hydrophobic interaction between the hydrophobic surface of NFs and the hydrophobic tail of lipids. In a previous study, we had proposed the structures of NFs, as shown in Figure 1b, based on various structural analyses including wide-angle X-ray diffraction (WAXD), small-angle X-ray scattering (SAXS), Fourier-transform infrared spectroscopy (FT-IR), circular dichroism (CD), transmission electron microscopy (TEM), and atomic force microscopy (AFM) [39]. β-sheet structures consisting mainly of hydrophobic amino acids formed a NFs framework with EG chains, providing water dispersibility. Thus, the surfaces of NFs had hydrophobic and hydrophilic domains (Figure 1b). Based on our proposed NFs model, the hydrophobic interactions between NFs and phospholipids would have been more effectively inhibited by longer EG chains, which may explain the EG length-dependence of the interactions between DPPC and NFs observed herein (using the Langmuir monolayer). However, because the building-block molecule of NFs has three carboxylic acids (aspartic acid residue, glutamic acid residue, EG-terminal) and its net charge under neutral conditions is minus 1, the possible contribution of electrostatic interactions between the negatively charged amino acid of the NFs and the quaternary-ammonium ion of DPPC cannot be ruled out. Direct observation of the DPPC/NF membrane with AFM and Brewster angle microscopy would confirm the location of NFs interactions with the DPPC membrane (inside or at the surface). Because NFs are surface active and adsorb at the air–water interface, there is possibility that NFs themselves form film by compression even without DPPC. The comparison of the DPPC/NFs film to the sole NFs film would be helpful to understand the interaction mechanism of NFs with the DPPC membrane. In addition, surface potential measurements of DPPC/NF membrane also provide deeper insight to the binding manner of NFs to DPPC membrane.

No significant difference was observed depending on the EG chain length in the changes of π with time after peptide addition (Figure 6), or in the π–*A* measurements in the presence of the peptides (Figure 7 and Figure 8). The addition of EG_n_ peptides to the subphase increased the π value irrespective of the EG length, unlike the behavior for the EG_n_ NFs systems (Figure 6). This may be explained because monomeric peptides more easily incorporated into the DPPC membrane, owing to their small size compared to those of the EG_n_ NFs. The π–*A* isotherm curves of DPPC in the presence of EG_n_ peptides were dependent on the peptide concentration. At a higher concentration (1.0 μM), the π–*A* isotherm curves of the binary systems were completely different from those of the sole DPPC membrane (Figure 7). It has been reported that β-sheet peptides themselves form a monolayer at the air–water interface by compression [45,46]. π was already high at the start point of compression, which indicated that a large amount of EG_n_ peptides might be present at the interface. Thus, the monolayer composed of EG_n_ peptides might have formed by compressing the EG_n_ peptides at the interface, which could be the reason for the difference in the isotherms for the EG_n_ peptides/DPPC and the sole DPPC membrane. In contrast, at lower concentrations (0.1 μM), the π–*A* isotherms of the binary systems were quite similar to that of the sole DPPC membrane, although a slight shift toward higher *A* was observed. Moreover, in the region of higher π, the curves of the binary systems almost overlapped with those of the sole DPPC membrane, which demonstrated the squeezing out of EG_n_ peptides to the subphase. These results indicated that nanofiber formation of EG_n_ peptides significantly change their interactions with phospholipids.

In a previous study, we reported that EG_n_ NFs with shorter EG chains were more efficiently associated with JAWS II cells and exhibited higher cytotoxicity [40]. The EG-length dependency for cellular interaction had the same tendency as that for the interaction with the DPPC membrane. Thus, the interaction of EG_n_ NFs with phospholipid membranes can be a crucial factor on determining the cellular association and toxicity of NFs, although several differences between the actual cell membrane and the DPPC membrane existed. In addition, we may derive that the interactions of NFs with cellular membrane can be tuned by the EG length, which facilitates the optimization of their properties as an antigen delivery carrier.

## 4. Materials and Methods

### 4.1. Materials

21-amino-*N*-(9-fluorenylmethoxycarbonyl)-4,7,10,13,16,19-hexaoxaheneicosanoic acid (Fmoc-*N*-amido-dPEG_6_ acid), 39-amino-*N*-(9-fluorenylmethoxycarbonyl)-4, 7, 10, 13, 16, 19, 22, 25, 28, 31, 34, 37-dodecaoxanonatriacontanoic acid (Fmoc-*N*-amido-dPEG_12_ acid), and O-[*N*-(9-fluorenylmethoxycarbonyl)-2-aminoethyl]-O’-(2-carboxyethyl) undecaethyleneglycol (Fmoc-*N*-amido-dPEG_24_ acid) were purchased from Quanta BioDesign Ltd. (Plain City, OH, USA). 2-chlorotrityl chloride resin, *N*,*N*’-diisopropylethylamine (DIPEA), all the L-Fmoc amino acids, 1-[bis(dimethylamino)methylene]-1H-benzotriazolium 3-oxide hexafluorophosphate (HBTU), 1-hydroxybenzotriazole (HOBT), and piperidine were purchased from Watanabe Chemical Industries Ltd. (Hiroshima, Japan). *N*,*N*’-dimethylformamide (DMF), isopropanol, methanol, diethyl ether, hexafluoroisopropanol (HFIP), dichloromethane (CH_2_Cl_2_), and trifluoroacetic acid (TFA) were purchased from Wako Pure Chemical Industries, Ltd. (Osaka, Japan). DPPC was purchased from Avanti Polar Lipids, Inc. (Alabaster, AL, USA).

### 4.2. Experimental Methods

#### 4.2.1. Synthesis of Building Block Molecules

EG_n_ peptides (*n* = 6, 12, and 24) were prepared using L-Fmoc amino acids and PEG that having carboxyl group and Fmoc-protected amino group at their terminal, Fmoc-*N*-amido-dPEG_n_ acid, by Fmoc solid-phase peptide synthesis, according to our previous reports [40]. Molecular weight was analyzed by MALDI-TOF mass (autoflex speed system, Bruker, Billerica, MA, USA). MS (MALDI-TOF): EG_6_; Cald. MASS: 2234.92, Obsd. MASS: 2234.359, EG_12_; Cald. MASS: 2499.12, Obsd. MASS: 2498.11, EG_24_; Cald. MASS: 3027.52, Obsd. MASS: 3027.04.

#### 4.2.2. Preparation of Antigen-Loaded Peptide NFs

The three types of NFs with different EG lengths were prepared by the previously reported procedure [40]. EG_n_ peptide was dissolved in HFIP and dried with N_2_ flow to allow for film formation. The obtained film was re-dissolved at a concentration of 1.5 mM in PBS. The solution was incubated at 60 °C for 24 h. Following incubation, the resulting peptide nanofiber dispersion was dialyzed against PBS for 24 h using a dialysis membrane (MWCO 14,000, GE Healthcare, Chicago, IL, USA) to remove free peptides. The length of NFs was controlled by filtration using a syringe filter with a pore size of 0.45 μm (GE Healthcare). To confirm the nanofiber formation, TEM) measurements were performed using a JEM-1200EX II (JEOL, Tokyo, Japan) with an acceleration voltage of 85 keV. The samples were negatively stained with 0.1% phosphotungstate. The lengths of 50 randomly selected fibers from TEM images were measured and the average fiber length and standard deviation were calculated. The average fiber lengths were estimated to be 280 ± 120 nm for EG_6_ NFs, 280 ± 100 nm for EG_12_ NFs, and 320 ± 80 nm for EG_24_ NFs.

#### 4.2.3. Interaction of EG_n_ NFs with the DPPC Membrane

The DPPC membrane was prepared using a Langmuir balance (KSV NIMA small Minimicro 2, KSV instruments, Helsinki, Finland). Prior to the experiments, the trough and barriers were thoroughly cleaned with running water. The temperature of the Langmuir trough was 20–21 °C during the measurements. A total of 0.34 mM DPPC solution (29 μL) was added to a subphase consisting of 39 mL of PBS in the trough. It was left for 20 min to evaporate the solvent. The barriers of balance were compressed until the π value in the membrane reached 30 mN/m. EG_n_ NFs suspension was injected slowly into the buffer subphase below the membrane at a speed of 6 mL/h using a syringe pump (KDS-100, KD Scientific, Holliston, MA, USA) to avoid disturbing the membrane. Changes in π of the DPPC membrane were recorded as a function of time. Representative data from at least several times repeats are shown.

#### 4.2.4. Compression Isotherm of DPPC in the Presence of EG_n_ NFs

The π–*A* isotherm measurements were performed using a Langmuir balance. The temperature of the Langmuir trough was 20–21 °C during the measurements. Each experiment was repeated, and representative data were reported. DPPC was dissolved in CHCl_3_ at a concentration of 0.34 mM. To obtain the π–*A* isotherm, approximately 29 μL of the DPPC solution was added dropwise onto the surface of PBS in the trough using a microsyringe. After waiting for 10 min to allow for solvent evaporation, a dispersion of NFs was added at a final concentration of 1.0 and 2.5 μM. After kept for 20 min, the barrier was compressed at a speed of 2 mm/min until the membrane collapsed. The measurements were repeated at least three times for each sample.

## 5. Conclusions

In this study, we investigated the effect of the interactions between three types of NFs with different EG lengths (6-, 12- and 24-mer) and dipalmitoylphosphatidylcholine (DPPC) Langmuir monolayers. The changes in π with time after the injection of EG_n_ NFs below the membrane differed significantly according to the EG length. The addition of EG_6_ NFs caused an increase in π in contrary to EG_12_ NFs and EG_24_ NFs. The π–*A* isotherms for the EG_24_ NFs/DPPC system were different from those for the other EG_n_ NFs/DPPC system, and almost overlapped with that for sole DPPC at high π, indicating little interaction between DPPC and EG_24_ NFs. In addition, the *ε*_0_–π plots revealed that the maximum *ε*_0_ for EG_6_ NFs/DPPC system was the smallest among the EG_n_ NFs/DPPC systems, indicating that the disturbing effect of EG_6_ NFs were the greatest. Based on the three types of systems, the interactions of NFs with the DPPC membrane were estimated to be higher with decreasing EG length. Because this trend was similar to the EG length dependency observed in cellular association and cytotoxicity of EG_n_ NFs, the interaction of EG_n_ NFs with phospholipid membranes can be a crucial factor to determine the cellular association and toxicity of NFs. In contrast to the NFs systems, no difference was observed because of EG-chain length variations on the interaction of the EG_n_ peptide with the DPPC membrane. This confirmed that nanofiber formation of EG_n_ peptides led to significant changes on their interactions with phospholipids. The results of this study may provide a molecular design guideline in β-sheet peptide nanofibrous formulation, and can be beneficial to expand its potential applications.

## Supplementary Materials

Supplementary materials can be found at https://www.mdpi.com/1422-0067/21/24/9518/s1, Figure S1: Changes in surface pressure of the DPPC membrane 30 min after injection of EG_n_ NFs.; Table S1: Collapse surface pressures, limiting molecular areas, and maximum elastic moduli determined from π-A isotherms.
