# Supplementary material for: Investigation on the Interactions between Self-Assembled β-Sheet Peptide Nanofibers and Model Cell Membranes
Source: Int J Mol Sci. 2020 Dec 14;21(24):9518. doi: 10.3390/ijms21249518 (PMC7765088; doi:10.3390/ijms21249518)
Supplement: Supplementary file 1 [file ijms-21-09518-s001.pdf]

Supplementary Material

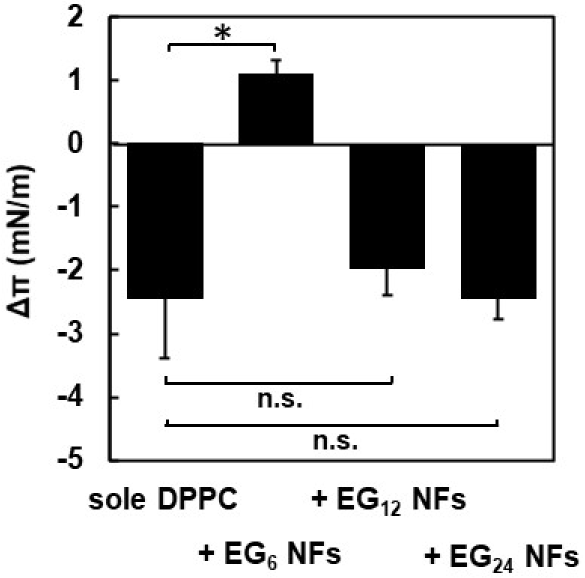

Figure 1. Changes in surface pressure of the DPPC membrane 30 min after injection of EG_n NFs. The $\Delta\pi$ was calculated by subtracting the surface pressure at 0 min from that at 30 min. The same measurements were repeated three times. The representative data are shown in Figure 3. **p* < 0.01, n.s. = not significant.

Table 1. Collapse surface pressures, limiting molecular areas, and maximum elastic moduli determined from π-A isotherms.[a]

	DPPC	EG_6 NFs		EG_{12} NFs		EG_{24} NFs	
		1.0 µM	2.5 µM	1.0 µM	2.5 µM	1.0 µM	2.5 µM
final collapse surface pressure (mN/m)	59.0	56.2	58.9	61.5	58.9	56.6	54.0
limiting molecular area at final collapse (Å^2)	19.8	18.6	18.2	24.6	20.1	23.4	26.5
maximum elastic moduli (mN/m)	137.5	66.2	48.4	68.7	63.1	59.8	65.0

[a] Collapse surface pressures, limiting molecular areas, and maximum elastic moduli were estimated from Figure 4 and 5.
